# Diarrheagenic *Escherichia coli* in Stool Specimens Collected from Patients Attending Primary Healthcare Facilities in Ethiopia: Whole-Genome Sequencing-Based Molecular Characterization

**DOI:** 10.3390/ijms251910251

**Published:** 2024-09-24

**Authors:** Deneke Wolde, Tadesse Eguale, Girmay Medhin, Aklilu Feleke Haile, Haile Alemayehu, Adane Mihret, Mateja Pirs, Katja Strašek Smrdel, Jana Avberšek, Darja Kušar, Tjaša Cerar Kišek, Tea Janko, Andrej Steyer, Marjanca Starčič Erjavec

**Affiliations:** 1 Department of Medical Laboratory Science, College of Medicine and Health Sciences, Wachemo University, Hossana P.O. Box 667, Ethiopia; denekewolde@gmail.com; 2 Aklilu Lemma Institute of Pathobiology, Addis Ababa University, Addis Ababa P.O. Box 1176, Ethiopia; tadesse.eguale@aau.edu.et (T.E.); girmay.medhin@aau.edu.et (G.M.); aklilu.feleke@aau.edu.et (A.F.H.); haile.alemayehu@aau.edu.et (H.A.); 3 Department of Microbiology, Biotechnical Faculty, University of Ljubljana, 1000 Ljubljana, Slovenia; 4 Ohio State Global One Heath, Addis Ababa P.O. Box 1176, Ethiopia; 5 College of Health Sciences, Addis Ababa University, Addis Ababa P.O. Box 1176, Ethiopia; amihret@gmail.com; 6 Armauer Hansen Research Institute, Addis Ababa P.O. Box 1005, Ethiopia; 7 Institute of Microbiology and Immunology, Faculty of Medicine, University of Ljubljana, 1000 Ljubljana, Slovenia; mateja.pirs@mf.uni-lj.si (M.P.); katja.strasek@mf.uni-lj.si (K.S.S.); 8 Institute of Microbiology and Parasitology, Veterinary Faculty, University of Ljubljana, 1000 Ljubljana, Slovenia; jana.avbersek@vf.uni-lj.si (J.A.); darja.kusar@vf.uni-lj.si (D.K.); 9 National Laboratory of Health, Environment and Food, 2000 Maribor, Slovenia; tjasa.cerar.kisek@nlzoh.si (T.C.K.); tea.janko@nlzoh.si (T.J.); andrej.steyer@nlzoh.si (A.S.)

**Keywords:** diarrheagenic *Escherichia coli*, whole-genome sequencing, phylogeny, virulence-associated genes, antibiotic resistance genes, Ethiopia

## Abstract

The diarrheagenic *Escherichia coli* (DEC) is the major cause of diarrheal diseases in Africa, including Ethiopia. However, the genetic diversity of *E. coli* pathotypes found in Ethiopia has not been studied well. This study aimed to characterize potential DEC belonging to enteropathogenic (EPEC), Shiga toxin-producing (STEC), enteroaggregative (EAEC), enterotoxigenic (ETEC), and enteroinvasive (EIEC) *E. coli* pathotypes from stool specimens of patients attending primary healthcare units (*n* = 260) in Addis Ababa and Hossana using whole-genome sequencing. Real-time PCR assays were used to identify DEC isolates belonging to EPEC, STEC, EAEC, ETEC, and EIEC pathotypes, which were then subjected to whole-genome sequencing on the Illumina platform. Twenty-four whole-genome nucleotide sequences of DEC strains with good enough quality were analyzed for virulence-associated genes (VAGs), antibiotic resistance genes (ARGs), phylogenetic groups, serogroups, and sequence types. The majority (62.5%) of DEC isolates belonged to the phylogenetic group B1. The identified DEC isolates belonged to 21 different serogroups and 17 different sequence types. All tested DEC isolates carried multiple VAGs and ARGs. The findings highlight the high diversity in the population structure of the studied DEC isolates, which is important for designing targeted interventions to reduce the diarrheal burden in Ethiopia.

## 1. Introduction

*Escherichia coli* is a genetically heterogeneous bacterium known to survive in various niches, including the gastrointestinal tract of humans and warm-blooded animals [1]. It is also one of the bacteria that poses a significant threat to human health due to its increasing resistance to different groups of antibiotics using a variety of mechanisms [2]. According to the World Health Organization (WHO), the resistance of *E. coli* to common antibiotics has reached alarming level in many parts of the world [3]. Furthermore, *E. coli* is known to serve as a reservoir for various antibiotic resistance genes (ARGs) and is capable of horizontally transferring these genes to other pathogenic and commensal organisms. Therefore, understanding the antimicrobial susceptibility of *E. coli* and genetic markers associated with resistance may provide an indication of the burden of antimicrobial resistance in other Gram-negative organisms circulating in a given community [4]. Moreover, antimicrobial resistance in *E. coli* strains is rapidly increasing in Ethiopia [5]. Multidrug resistance was reported in 62–88% of *E. coli* isolates in this country [6,7,8].

Some strains of *E. coli* harbor virulence factors that affect a broad spectrum of cellular functions and lead to a variety of intestinal and extraintestinal infections, including diarrheal diseases, neonatal meningitis, septicemia, and urinary tract infections [9]. Diarrheagenic *E. coli* (DEC) strains have been classified by their virulent characteristics into six different pathotypes: enteropathogenic *E. coli* (EPEC), enterotoxigenic *E. coli* (ETEC), enteroinvasive *E. coli* (EIEC), enteroaggregative *E. coli* (EAEC), diffusely adherent *E. coli* (DAEC), and Shiga toxin-producing *E. coli* (STEC) [10]. These pathotypes carry one or more specific virulence genes that are not present in commensal *E. coli* [11]. The genes encoding virulence factors in *E. coli* are likely acquired through horizontal gene transfer from other bacterial species through plasmids, integrons, bacteriophages, pathogenicity islands, and transposons [12]. The most prominent and well-characterized pathotypes known to cause diarrhea are EPEC, ETEC, EAEC, STEC, and EIEC [13]. However, the pathogenicity and epidemiological importance of DAEC isolates in causing diarrheal disease has been the subject of ongoing debate and controversy [14]. DEC pathotypes are responsible for about 30–40% of acute diarrhea episodes in children [15]. They also play a considerable role in causing diarrhea in adults [16]. They are widespread worldwide, with prevalence varying by geographic region [17] and the incidence of different pathotypes in different age groups [18,19]. The variability in distribution of DEC is also observed in Ethiopia. For example, a study conducted in central Ethiopia and Wolaita Sodo, southern Ethiopia, found a high incidence of the EAEC strain [6,20]. In contrast, a study conducted in Bahir Dar town in northwestern Ethiopia showed a high prevalence of ETEC in children and calves [21]. On the other hand, a study conducted in the northwestern region of Ethiopia reported a high prevalence of the STEC strain from abattoir workers hands, carcass, cattle feces, and other environmental samples [22].

Phylogenetic grouping and sequence typing are used to categorize different strains of *E. coli* based on their genetic relatedness. *E. coli* can be assigned into different phylogroups (e.g., A, B1, B2, C …) based on the detection of the gene encoding the ankyrin repeat protein A (*arpA)*, a gene required for heme transport *(chuA)* and a gene that encodes a putative transcriptional regulator *(yjaA)* and the DNA fragment TSPE4.C2 [23]. Multilocus sequence typing is the genotyping method based on the sequences of specific loci of seven housekeeping genes that are highly conserved and present in all *E. coli* strains. Isolates are assigned into different sequence types (STs) based on the combination of different alleles of the seven housekeeping genes for each strain [24].

Genome analysis of the DEC strains can provide a comprehensive overview of the distribution of different DEC pathotypes. This analysis can reveal the repertoire of virulence-associated genes (VAGs) and ARGs present in DEC isolates. Furthermore, whole-genome sequence-based phylogenetic analysis can help clarify the evolutionary relationship and genetic relatedness between the different DEC strains [25]. This information can then be utilized for public health investigations, such as tracing the source and transmission patterns of DEC strains, identifying the emergence and spread of new, potentially more virulent or antibiotic-resistant DEC strains, and monitoring the evolution and dissemination of DEC subtypes across different geographic regions and populations. However, there is a lack of comprehensive genomic data on the diversity of *E. coli*, as well as the repertoires of ARGs, VAGs, STs, and serotypes. Therefore, the objective of this study was to characterize DEC isolates (VAGs, ARGs, STs, serotypes, and phylogenetic groups) of the most prominent pathotypes (EPEC, STEC, EAEC, ETEC, and EIEC) obtained from stool samples of patients visiting primary healthcare facilities in Addis Ababa and Hossana, Ethiopia, using whole-genome sequencing and bioinformatic tools.

## 2. Results

### 2.1. Whole-Genome Sequencing of DEC Isolates

All 30 DEC isolates detected by real-time PCR were subjected to whole-genome sequencing (WGS). Based on the preliminary quality assessment criteria, which included a depth of coverage > 30×, N50  >  15 kbp, and <500 contigs in the assembly, whole-genome sequence data of only 25 isolates passed and were hence further used for bioinformatics analysis. The whole-genome sequence analysis revealed that one out of the 25 isolates did not harbor any DEC specific genes, so whole-genome sequence data from this isolate were excluded from further analysis. The predominant DEC pathotype was the EPEC strain (10/24; 41.7%), followed by the EAEC (6/24; 25%) and ETEC strains (5/24; 20.8%). Three (12.5%) of *E. coli* isolates belonged to STEC pathotype. No *E. coli* isolates belonged to EIEC pathotype. All STEC isolates were detected in stool from non-diarrheic patients; however, the other pathotypes were detected from both diarrheic and non-diarrheic patients. Of the 24 DEC isolates, 14 (58.3%) were isolated from stool specimens of diarrheic patients. Overall, the DEC isolates were detected in equal frequency in both Addis Ababa and Hossana. A high proportion of DEC isolates (45.8%) was detected in the age group 20–45 years. However, there was no significant difference in the distribution of DEC among different age groups (Fisher exact *p* = 0.873). The prevalence of DEC isolates was slightly higher in females (58.3%) compared to males (41.7%) (Table 1).

According to the whole-genome sequence analysis, the Shiga toxin 1 encoding gene (*stx1)*, with its subtypes *stx1c* and *stx1a*, was detected in two isolates, while the Shiga toxin 2 encoding gene (*stx2)* variant *stx2c* was detected in one isolate. Six different subtypes of intimin (*eaeA)* genes (*eae-a01-α*, *eae-b01a-β*, *eae-e02-ε*, *eae-e08-π*, *eae-g02-θ*, and *eae-e06-η*) were detected in EPEC isolates. Furthermore, two variants of heat-labile enterotoxins (*eltIAB-8* and *eltIAB-11*) and one variant of heat-stable enterotoxin (*estah-STa3*), specific to ETEC, were detected (Table 2).

### 2.2. Serotypes, Phylogenetic Groups, and Multilocus Sequence Typing of DEC Isolates

The DEC isolates (*n* = 24) confirmed by WGS belonged to 21 different serotypes. Five isolates (20.8%) belonging to STEC, EPEC, and ETEC pathotypes were defined as ‘rough’ (lacking expression of the O antigen). Serotyping results showed that the EPEC isolates belonged to nine different serotypes (Table 2 and Table S1).

**Figure 1:**
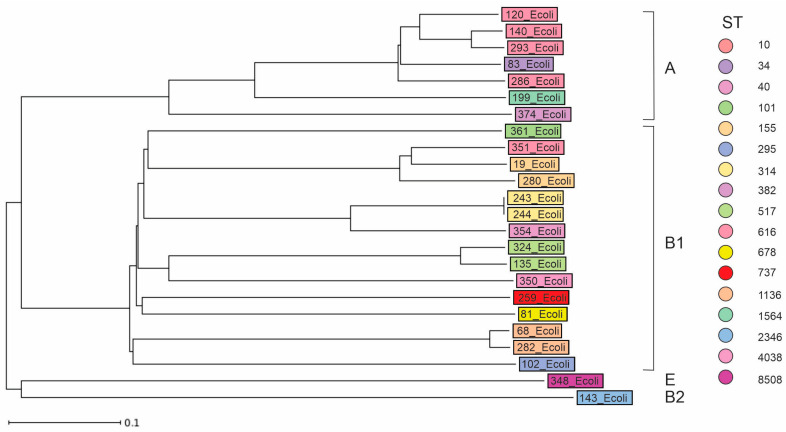
Phylogenetic tree of 24 WGS-confirmed DEC isolates. Different sequence types (STs) are color marked. The phylogenetic groups of the isolates are also given.

Phylogenomic analysis revealed considerable diversity among the 24 WGS confirmed DEC isolates. Most of the isolates (*n* = 15; 62.5%) belonged to phylogenetic group B1, seven (29.2%) to group A, and the remaining isolates to phylogroups B2 (*n* = 1; 4.2%) and E (*n* = 1; 4.2%) (Figure 1).

Phylogroup B1 was equally detected in Hossana and Addis Ababa, while four isolates from Addis Ababa and three isolates from Hossana were detected in phylogroup A. Phylogroup B2 was detected only among isolates from Hossana. Phylogroups A and B1 were detected in all age groups except in the age group 5–9 years. ‘Rough’ serotypes were identified as phylogroup A and B1 (Table S1).

The 24 WGS-confirmed DEC isolates were assigned to 17 different STs, of which the dominant DEC pathotype (EPEC) was detected in isolates belonging to 8 different STs and 9 different serotypes. The most frequent ST was the ST10, representing four (16.7%) of the DEC isolates (Table 2).

### 2.3. Virulence-Associated Genes of DEC Isolates

The VAGs profiles of each *E. coli* isolates are summarized in Table S1. Among 24 WGS-confirmed DEC isolates, 117 VAGs (involved in iron acquisition, adherence, and toxin production) were identified, of which 42 VAGs were detected only in a single pathotype. All DEC isolates tested had 14 to 37 VAGs. Virulence-associated genes were detected more frequently in the EPEC strain compared to other pathotypes. Genes involved in toxin production, including Shiga toxin-encoding genes (*stx1* or *stx2*) and enterotoxin-encoding genes, such as *astA*, *eltIAB-8*, *eltIAB-11*, *estah-STa3*, *estY2*, *pet*, *pic*, *sat* and *senB* were detected. In addition, bacteriocins and microcins encoding genes, namely *cba*, *cea*, *cia*, *cib*, *cma*, *colE2*, *colE5*, and *cva*, were detected in various DEC isolates. Other VAGs were also identified, including *ehxA*, encoding enterohemolysin, and *cdt-IIIB*, which is a variant of cytolethal distending toxin IIIB. In addition, phages associated with the *stx* genes were identified in STEC isolates using PHASTEST. This showed that the *stx1* gene was carried by a complete, intact phage in isolate 259, while the *stx2* gene was associated with an incomplete phage in isolate 280. In the third STEC isolate, isolate 361 possessing *stx1* gene, no phage sequences in the contig harboring the *stx1* gene were detected, although the contig was long enough. Furthermore, the cytotoxic necrotizing factor 2 (*cnf2*), which is associated with cell death or tissue damage, which is a defining virulence factor of necrotoxigenic *E. coli* (NTEC2), was detected in the isolate 361. This suggests that this isolate represents a hybrid pathotype, containing virulence factors associated with both NTEC and STEC strains (Table 3 and Table S1).

Numerous VAGs were identified in all phylogroups, with the genes being more common in phylogroup B1 strains than in strains from other groups. Phylogroup A and B1 shared 52 similar virulence-associated genes. Additionally, seventeen different virulence-associated genes were detected in phylogroup B2 strain. Two virulence-associated genes (*eilA* and *espY2*) were exclusively detected in phylogroup E. Eight virulence-associated genes such as *hlyE*, *nlpI*, *lpfA*, *terC*, *yehA*, *yehB*, *yehC*, and *yehD* were identified in the entire phylogroup B1. The *toxB* and *espC* genes were detected only in DEC isolate of phylogroup B2. Five virulence-associated genes, namely, *nlpI*, *terC*, *yehB*, *yehC*, and *yehD*, were confirmed to be shared by all four phylogroups (Table 4 and Table S1).

### 2.4. Antimicrobial Resistance Genes in DEC Isolates

In the phenotypic antimicrobial susceptibility testing, 18 out of the total 30 DEC isolates (60%) showed resistance to at least one of the tested antimicrobial agents (Wolde et al., 2024). A total of 23 different ARGs conferring resistance to β-lactams, aminoglycosides, quinolones, sulfonamides, trimethoprim, the macrolide/lincosamide/streptogramin (MLS) group, and tetracycline were detected in 18 (75%) DEC isolates out of 24 isolates subjected to whole-genome sequencing (Table 5). The most common ARG was *bla*_TEM-1B_, which was found in 12 DEC isolates. The results indicated that the EPEC and ETEC isolates carried multiple ARGs conferring resistance to a range of antimicrobials. In contrast, the STEC isolates were not resistant to any of the tested antibiotics and did not harbor any of the ARGs.

### 2.5. Plasmids and Other Mobile Genetic Elements

**Figure 2:**
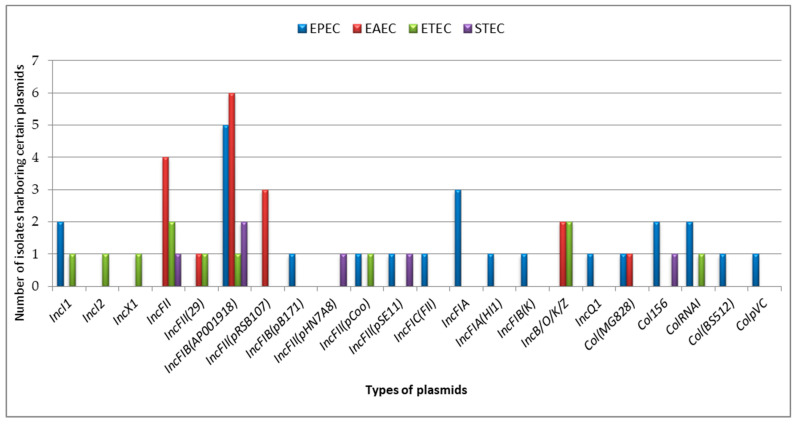
Frequency of plasmid types in pathotypes of studied DEC isolates.

Eighty-two different mobile genetic elements were identified in *E. coli* isolates in this study. Of these, 22 were plasmids, 50 were insertion sequence elements (ISs), 9 were transposons (three-unit transposons and six composite transposons), and 1 was a miniature inverted repeat (*MITEEc1*). The majority of MGEs were detected in the EPEC strain, followed by the EAEC strain. Twenty-three MGEs (9 plasmids, 10 ISs, and 4 transposons) were identified only in the EPEC isolates. Twenty-two (91.7%) of the DEC isolates were confirmed to harbor one or more plasmids. The *IncF* plasmid with different replicon types and replicon variants, including *IncFII*, *IncFII(29)*, *IncFIB(AP001918)*, *IncFII(pRSB107)*, *IncFIB(pB171)*, *IncFII(pHN7A8)*, *IncFII(pCoo)*, *IncFII(pSE11)*, *IncFIC(FII)*, *IncFIA(HI1)*, *IncFIA*, and *IncFIB(K)* was the most abundant plasmid in *E. coli* isolates. Additionally, *Col* plasmid groups such as *Col(MG828)*, *Col156*, *ColRNAI*, *Col(BS512)*, and *ColpVC* were also detected in DEC isolates. Fifteen (68.2%) of the total plasmids were identified in EPEC isolates, while only five (22.7%) were identified in STEC isolates (Figure 2).

In the current study, we found that all DEC isolates harbored *MITEEc1*, 66.7% harbored *IS609*, and 62.5% harbored *ISEc1*. Transposons such as *Tn2*, *Tn7*, and *Tn6024* were identified in 16.7% of the DEC isolates. Thirty-seven (61.7%) of the MGEs, apart from plasmids, were detected in the EPEC strain, while 29 (48.3%) were detected in the ETEC strain.

**Figure 3:**
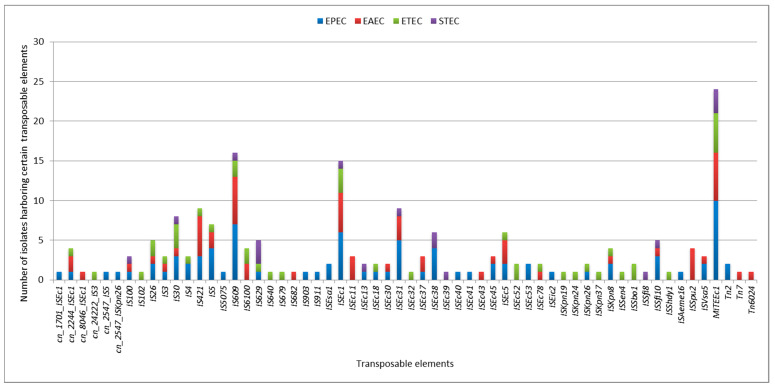
Frequency of transposable elements in pathotypes of studied DEC isolates.

On average, all the DEC isolates carried 7 MGEs, excluding plasmids, with some carrying as many as 16. *MITEEc1* and 11 IS elements, including *IS100*, *IS30*, *IS609*, *IS629*, *ISEc1*, *ISEc13*, *ISEc31*, *ISEc38*, *ISEc39*, *ISSfl8*, and *ISSfl10*, were detected in the STEC isolates (Figure 3).

## 3. Discussion

The DEC strains exhibited genetic diversity both within and between different pathotypes [26]. The overall rate of DEC isolation in this study was 11.5%, which is comparable to previous reports in diarrheic children under the age of five years and in tracked human contacts in rural and urban areas in Eastern Ethiopia (10.3%) [27], among diarrheic children in Abuja, Nigeria (12.8%) [28], as well as in children with diarrhea in Burkina Faso (7.4%) [29]. However, a higher number of DEC isolates were reported in diarrheic patients of all age groups in Kwali, Nigeria (27%) [30], and in Tunisia among children and adults with and without diarrhea (48.2%) [31]. Similarly, high rates of DEC isolates were detected in children with and without diarrhea in central Ethiopia (38.2%) [20], in Nigeria (73.8%) [32], and in Gabon (68.5%) [33], in children under five years with diarrhea and food animals in Kenya (23.0%) [34] and in Mozambique (48.6%) [35].

Phylogenetic group analysis of unknown *E. coli* strains is crucial for understanding the population structure and diversity of *E. coli* strains [36]. The present study showed a difference in phylogroup structure, with phylogroup B1 being predominant (62.5%), which is consistent with a study conducted in southwestern Nigeria among *E. coli* isolates from human where phylogroup B1 was predominant [37]. In contrast, a study conducted among diarrheic children and adults in Cote d’Ivoire showed the predominance of phylogroup A (53%) [38]. In addition two studies on *E. coli* isolated from different clinical sources in Egypt from patients aged 10 to 65 years and symptomatic UTIs (urinary tract infections) and diarrheic patients showed the predominance of phylogroup A (53%) [39,40].

*E. coli* strains belonging to phylogroup A and B1 are commonly considered as commensal organisms [41]. However, this study found that *E. coli* strains in these groups contained a high number of VAGs that enable them to cause infection in the intestinal tract, which may have been acquired through horizontal gene transfer [42], representing a significant health concern. This is consistent with the finding of a systematic review and meta-analysis of human commensal *E. coli* from different regions of the world that showed dominance of phylogroup A and B1 [36].

This is the first study in Ethiopia to determine the population structure of DEC isolates using WGS, providing a comprehensive understanding of the genetic diversity of DEC isolates. The DEC isolates in our study revealed high genetic diversity in the population structure of DEC pathotypes. This is in agreement with a study conducted in Malawi using *E. coli* collected from 2012 to 2018 [43]. Seventeen different STs were identified in our study, with the dominant EPEC pathotype in this study belonging to eight different STs and nine different serotypes, indicating a broad distribution of genes encoding the EPEC strain across the different STs and serotypes of *E. coli*. Each ST contained a variety of VAG combinations. This poses a challenge in reducing the transmission of infections associated with DEC pathotypes. The most frequent ST was ST10. In a study conducted on EAEC isolates from children with and without diarrhea in Nigeria, ST10 was the most prevalent [44]. Our study, however, indicated that only one EAEC was in ST10.

Both commensal and pathogenic *E. coli* can share virulence factors that are essential to survive and colonize specific ecological niches in the host, highlighting the versatility of *E. coli* [45]. Our analysis revealed that all *E. coli* isolates tested carried a variety of VAGs encoding adhesins, toxins, protectins, and iron uptake factors. Genes related to extra-intestinal *E. coli* pathotypes, such as *csgA*, *fimH*, *hlyE*, *iss*, *nlpI*, *terC* and *traT*, were distributed across all pathotype groups. Of these, the genes *csgA*, *fimH*, and *traT* were detected in *E. coli* from stool, urine, and blood samples in Egypt [46]. The *fimH*, *afa*, *hly*, and *cnf* genes were also detected in *E. coli* isolated from patients with urinary tract infections in Addis Ababa, Ethiopia [47], and Tunisia [48]. In addition, the *fimH*, *vat*, *sitA*, *hlyF*, and *iutA* genes were detected in uropathogenic *E. coli* isolated from patients with suspected UTIs in Zimbabwe [49]. This suggests that *E. coli* strains carrying genes responsible for the development of both intestinal and extraintestinal infections may be derived from commensal strains through the acquisition of different virulence factors via horizontal gene transfer during their presence in the intestine, enabling them to become virulent and theoretically cause both intestinal and extraintestinal infections [45,50,51].

Hybrid pathotypes have diverse and potentially enhanced pathogenicity potential. The identification of such hybrid strains is an important public health concern [52]. In the current study, an NTEC/STEC hybrid pathotype was detected. This pathotype carried the *cnf2* gene, which encodes the cytotoxin necrotizing factor 2 and the *stx1* gene, which encodes for the Shiga toxin. Cytotoxic necrotizing factor and Shiga toxin are cyclomodulins known to modulate cellular differentiation, apoptosis, and proliferation [53]. The *cnf2* gene, which is known to encode a lethal and dermonecrotic toxin, is located on pVir-like conjugative plasmids [54]. The other cyclomodulin encoding gene detected in this isolate, the *cdt-IIIB* gene is also known to be located on pVir-like conjugative plasmids. This cyclomodulin induces DNA damage and cell cycle arrest, leading to cytotoxicity and facilitating the entry of other toxins [55]. The isolate also carried a serine protease encoded by *espP*, which has proteolytic activity and can cleave human coagulation factor V [56]. The synergistic effect of several toxins could increase the ability of the isolate to cause more severe disease. This pathotype can be considered the first to be reported from Ethiopia. This is an indication that appropriate prevention and control measures need to be developed and implemented to control this type of virulent strains as they may pose a potential health threat to the population.

The most widely used class of antibiotics in humans for the treatment of pathogenic *E. coli* is the β-lactams [57]. However, in this study, a high frequency of DEC isolates resistant not only to β-lactam but also aminoglycosides, quinolones, macrolides, sulfonamides, trimethoprim, and tetracycline antibiotics were found. The *bla*_TEM-1B_ gene was the dominant resistance gene among the DEC in our study. Similar results were found in a study in children under five years of age with and without diarrhea in Ethiopia [58], diarrheic patients in Ghana [59], and a systematic review and meta-analysis of *E. coli* isolates from human, animal, and environmental samples [60].

Several VAGs and ARGs are located on MGEs. The analysis of MGEs showed DEC isolates harbored different plasmids and other MGEs, which might indicate potentials for horizontal gene transfer, which can spread antibiotic resistance and virulence traits between bacterial populations [61].

STEC is a zoonotic pathogen and poses a major public health challenge due to its high pathogenicity for humans. It causes diarrhea, hemorrhagic colitis (HC), and hemolytic uremic syndrome (HUS) in humans worldwide [62]. In the current study, 12.5% of DEC isolates belonged to the STEC group. However, the underestimation or misclassification of STEC strains into different pathotypes could occur due to the loss of Stx-converting bacteriophages [63]. Importantly, all STEC isolates were sensitive to all antimicrobials tested and did not carry ARGs. This finding may be due to the fact that STEC infections are not usually treated with aggressive antimicrobial therapy [64,65]. Despite the relatively low proportion of STEC strains among DEC isolates and the absence of antimicrobial resistance, the development of appropriate risk mitigation strategies, such as implementing strict hygiene and sanitation practices in food production, processing, and handling, as well as improving drinking water safety and environmental sanitation, is essential considering the significant impact that STEC infections can have on public health, as STEC infection is acquired via the fecal–oral route through contaminated food and water or direct contact with STEC-carrying animals [66]. The development of rapid and accurate detection methods for STEC strains is of great importance for improving prognosis and reducing mortality and associated complications [67].

The results of this study showed high diversity in the population structure of DEC isolates in terms of phylogroup, serotypes, sequence types, and VAGs. Serotypes that have higher potential to cause severe disease were also identified in this study.

## 4. Materials and Methods

### 4.1. Bacterial Isolates

A total of 260 *E. coli* isolates obtained from stool of (139 diarrheic and 121 non-diarrheic) patients of all age groups attending primary health care facilities in Addis Ababa and Hossana were used for the present investigation as a continuation of a previous study. As described in our previous study, *E. coli* was isolated from stool sample pre-enriched in buffered peptone water (BPW) and incubated overnight at 37 °C. Then, the enriched sample was streaked on eosin methylene blue agar (EMB), and colonies exhibiting a metallic green sheen were further examined using biochemical tests and confirmed by matrix-assisted laser desorption ionization time of flight (MALDI-TOF) mass spectrometry [68].

### 4.2. DNA Extraction and PCR Detection of E. coli Pathotypes Genes

Genomic DNA was extracted using STARMag 96 × 4 Universal Cartridge Kit (Seegene Inc, Walnut Creek, CA, USA) on the automated system Seegene STARlet (Seegene) according to manufacturer’s instructions. Multiplex real-time PCR was used to determine the presence of EPEC (*eaeA*), STEC (*stx1/stx2*), EAEC (*aggR*), and ETEC (*lt/st*) genes. The PCR reaction was prepared instantly on the same automated system Seegene STARlet (Seegene) according to the manufacturer’s instructions (Allplex^TM^ GI-Bacteria (II) Assay, Seegene). Briefly, the 25 μL of PCR reaction contained 5 μL 5× MuDT Oligo Mix (amplification and detection reagent), 10 μL of RNase-free water, 5 μL of premix with DNA polymerase, Uracil-DNA glycosylase (UDG), and Buffer containing dNTPs and 5 μL of DNA template. As a negative control, 5 μL of RNase-free water was used, whereas 5 μL of GI-B(II) PC was used as a positive control. The amplification and detection procedures were carried out on a CFX96^TM^ Real-time PCR Detection System (CFX Manager^TM^ Software-IVD v1.6) with steps: 20 min at 50 °C, 15 min at 95 °C, followed by 45 cycles of 10 s at 95 °C, 1 min at 60 °C and 30 s at 72 °C [69]. The determination of enteroinvasive *E. coli* (EIEC) was performed by real-time PCR, namely, by detecting the invasion plasmid antigen H (*ipaH*) gene. The primers and probe targeting the *ipaH* gene were previously described by Wang et al. (2010). In brief, the 10 µL PCR reaction contained 1× TaqMan Universal PCR Master Mix (Applied Biosystems, USA), 1× assay mix (mixture of 500 nM of both PCR primers and 200 nM of TaqMan probe labeled with FAM dye), and 1 µL of DNA template. PCR amplification (2 min at 50 °C, 10 min at 95 °C, followed by 35 cycles of 15 s at 95 °C and 1 min at 60 °C) and detection were performed using the QuantStudio 5 real-time PCR system (Applied Biosystems, USA) [70].

### 4.3. Whole-Genome Sequencing, Raw Data Pre-Processing, De Novo Assembly and Quality Control

WGS was performed for all DEC isolates identified by GI-Bacteria (II) Assay and *ipaH* PCR. Genomic libraries were prepared using Illumina DNA Prep (Illumina, San Diego, CA, USA). Isolates were sequenced on the NextSeq 2000 system (Illumina) using 2 × 150 bp paired-end reads chemistry [71]. Trimmomatic was used to trim raw reads from adapter sequences and low quality reads [72]. The quality of both raw and trimmed reads was assessed using FastQC v0.11.9 [73]. Species identification was confirmed using KmerFinder v3.0.2 based on trimmed reads. Assembly of trimmed reads into contigs was done with SPAdes v3.15.3 [74] using the default Kmer values and the “-isolate” parameters. Quast v5.2.0 was used for quality assessment of the assemblies [75]. Assemblies with N50 < 15 Kb and > 500 contigs were excluded. To determine coverage of assembly, fastq files were converted into .bam using samtools. Subsequently, »samtools depth« was used to obtain base coverages, which were then averaged and reported.

### 4.4. Phylogenetic Groups, In Silico Multilocus Sequence Typing, and In Silico Serotyping

Phylogenetic groups of DEC isolates were determined using ClermonTyping 2.0.9 [76] available at http://clermontyping.iame-research.center/ (accessed on 5 June 2024). STs were determined by MLST based on the seven gene Achtman scheme using the website https://pubmlst.org/ (accessed on 5 June 2024) [77]. The assembled genomes were compared using a curated *E. coli* core genome MLST scheme available in SeqSphere+ (version 10.0.2, Ridom, https://www.ridom.de, accessed on 2 September 2024). A phylogenetic tree was constructed using the neighbor joining algorithm with the software’s default parameters [78]. Target genes (188 in total) that were not found in all of the 24 samples were excluded from the analysis. The detection of DEC VAGs was performed using VirulenceFinder 2.0 available at the www.genomicepidemiology.org (accessed on 15 June 2024) with the default parameters (90% identity over 60% minimum Query/high-scoring segment pair length) [79]. Gene prediction was confirmed if the assembled sequence had at least 97% nucleotide match and 100% coverage with genes in the curated *Escherichia coli* database. SerotypeFinder 2.0 available at the www.genomicepidemiology.org (accessed on 15 June 2024) was used to identify the serotypes of *E. coli* with the default parameters (85% identity over 60% minimum Template/HSP length) [80]. Mobile genetic elements were identified using MobileElementFinder version 1.0 with MGEdb v1.0.2, available at https://cge.food.dtu.dk/services/MobileElementFinder/ (accessed on 26 August 2024) [81]. The *stx*-carrying prophages from whole-genome sequenced STEC isolates were identified by using PHASTEST (phage search tool with enhanced sequence translation) web-based tool (https://phastest.ca/) (accessed on 3 September 2024) [82].

### 4.5. Statistical Analysis

Descriptive analyses were performed using Microsoft Excel. Frequency and percentages were used to summarize the variables. The Fisher exact test was used to assess association between distribution of DEC and different variables using the Stata 14.0. A *p*-value of < 0.05 was considered statistically significant.

## Figures and Tables

**Table 1:** Distribution among study sites and patient’s characteristics of DEC isolates.

Characteristics	Number of DEC Isolates (%)			
	EPEC (*n* = 10)	EAEC (*n* = 6)	ETEC (*n* = 5)	STEC (*n* = 3)
Study site				
Addis Ababa (*n* = 12)	4 (16.7)	3 (12.5)	2 (8.3)	3 (12.5)
Hossana (*n* = 12)	6 (25)	3 (12.5)	3 (12.5)	0
Sex				
Male (*n* = 10)	6 (25.0)	3 (12.5)	0	1 (4.2)
Female (*n* = 14)	4 (16.7)	3 (12.5)	5 (20.8)	2 (8.3)
Participants				
Diarrheic (*n* = 14)	7 (29.2)	3 (12.5)	4 (16.7)	0
Non-diarrheic (*n* = 10)	3 (12.5)	3 (12.5)	1 (4.2)	3 (12.5)
Age group				
0–4 (*n* = 3)	2 (8.3)	0	1 (4.2)	0
5–9 (*n* = 2)	1 (4.2)	1 (8.3)	0	0
10–14 (*n* = 3)	2 (8.3)	0	0	1 (4.2)
15–19 (*n* = 2)	0	1 (4.2)	1 (4.2)	0
20–45 (*n* = 11)	5 (20.8)	3 (12.5)	2 (8.3)	1 (4.2)
46–65 (*n* = 3)	0	1 (4.2)	1 (4.2)	1 (4.2)

**Table 2:** Features of DEC isolates in terms of phylogroup, sequence types (STs), serotypes, and pathotype-specific virulence gene distribution.

Pathotype as Determined by WGS	Phylogroup	ST	Serotype	Pathotype Specific Virulence Genes
EPEC	A	10	O90:H40	*eae-g02-θ*
	A	10	O90:H40	*eae-g02-θ*
	A	382	H5	*eae-e08-π*
	B1	40	O21:H21	*eae-b01a-β*
	B1	517	O111:H19	*eae-e02-ε*
	B1	517	H19	*eae-e02-ε*
	B1	616	H21	*eae-e06-η*
	B1	4038	O93:H28	*eae-e06-η*
	B2	2346	O142:H34	*eae-a01-α*
	E	8508	O136:H49	*eae-g02-θ*
EAEC	A	10	O65:H12	*aggR*
	A	34	O99:H33	*aggR*
	B1	295	O171:H5	*aggR*
	B1	678	O104:H4	*aggR*
	B1	1136	O59:H19	*aggR*
	B1	1136	O167:H19	*aggR*
ETEC	A	10	O159:H21	*estah-STa3*
	A	1564	H21	*estah-STa3*
	B1	155	O58:H51	*eltIAB-8*
	B1	314	O112ab:H21	*eltIAB-11*
	B1	314	O112ab:H21	*eltIAB-11*
STEC	B1	101	O117:H12	*stx1* (*stx1a*)
	B1	155	H25	*stx2* (*stx2c*)
	B1	737	O81:H21	*stx1* (*stx1c*)

**Table 3:** Distribution of VAGs among different DEC isolates as determined by WGS.

Virulence-Associated Genes and Their Encoded Proteins/Functions			Prevalence in Each Pathotypes N (%), N = 24			
			EPEC (*n* = 10)	EAEC (*n* = 6)	ETEC (*n* = 5)	STEC (*n* = 3)
Adhesins	*aap*	Dispersin, antiaggregation protein	0	6 (25)	0	0
	*afaA*	Transcriptional regulator	1 (4.2)	0	0	0
	*afaC*	Outer membrane usher protein	1 (4.2)	0	0	0
	*afaD*	Afimbrial adhesion	1 (4.2)	3 (12.5)	0	0
	*agg3A*	AAF/III major fimbrial subunit	0	3 (12.5)	0	0
	*agg3B*	AAF/III minor adhesin	0	3 (12.5)	0	0
	*agg3C*	Usher, AAF/III assembly unit	0	3 (12.5)	0	0
	*agg3D*	Chaperone, AAF/III assembly unit	0	3 (12.5)	0	0
	*agg4A*	AAF/IV major fimbrial subunit	0	2 (8.3)	0	0
	*agg4C*	Usher, AAF/IV assembly unit	0	2 (8.3)	0	0
	*aggA*	AAF/I major fimbrial subunit	0	1 (4.2)	0	0
	*aggC*	Usher, AAF/I assembly unit	0	1 (4.2)	0	0
	*aggD*	Chaperone, AAF/I assembly unit	0	1 (4.2)	0	0
	*aggR*	AraC transcriptional activator	0	6 (25)	0	0
	*cif*	Type III secreted effector	4 (16.7)	0	0	0
	*csgA*	Curli major subunit CsgA	9 (37.5)	6 (25)	5 (20.8)	2 (8.3)
	*cswR*	CS12 Transcriptional activator	0	0	2 (8.3)	0
	*eaeA*	Intimin	10 (41.7)	0	0	0
	*efa1*	EHEC factor for adherence; lymphostatin Efa1-LifA	1 (4.2)	0	0	0
	*etpB*	EtpB Nonfimbrial adhesin/TPS transporter	0	0	1 (4.2)	0
	*faeC*	F4 (K88) Minor fimbrial subunit	0	0	0	1 (4.2)
	*fdeC*	Intimin-like adhesin FdeC	5 (20.8)	2 (8.3)	3 (12.5)	3 (12.5)
	*fimH*	Type 1 fimbriae	8 (33.3)	5 (20.8)	4 (16.7)	3 (12.5)
	*hra*	Heat-resistant agglutinin	0	2 (8.3)	1 (4.2)	0
	*iha*	Adherence protein	0	2 (8.3)	0	3 (12.5)
	*lpfA*	Long polar fimbriae	4 (16.7)	4 (16.7)	3 (12.5)	3 (12.5)
	*nlpI*	Lipoprotein NlpI precursor	10 (41.7)	6 (25)	5 (20.8)	3 (12.5)
	*perA*	EPEC adherence factor	1 (4.2)	0	0	0
	*tir*	Translocated intimin receptor protein	9 (37.5)	0	0	0
	*yehA*	Outer membrane lipoprotein, YHD fimbrial cluster	6 (25)	3 (12.5)	4 (16.7)	2 (8.3)
	*yehB*	Usher, YHD fimbrial cluster	8 (33.3)	6 (25)	5 (20.8)	3 (12.5)
	*yehC*	Chaperone, YHD fimbrial cluster	8 (33.3)	6 (25)	5 (20.8)	3 (12.5)
	*yehD*	Major pilin subunit, YHD fimbrial cluster	8 (33.3)	5 (20.8)	5 (20.8)	3 (12.5)
	*yfcV*	Fimbrial protein	1 (4.2)	0	0	0
Protectins	*aaiC*	Type VI secretion protein	0	3 (12.5)	0	0
	*aar*	AggR-activated regulator	0	4 (16.7)	0	0
	*aatA*	Dispersin transporter protein	0	6 (25)	0	0
	*aamR*:FN554766	Not defined	0	1 (4.2)	0	0
	*anr*	AraC negative regulator	3 (12.5)	5 (20.8)	1 (4.2)	0
	*aslA*	Arylsulfatase-like protein	1 (4.2)	2 (8.3)	0	0
	*capU*	Hexosyltransferase homolog	2 (8.3)	3 (12.5)	1 (4.2)	0
	*cba*	Colicin B	0	0	0	1 (4.2)
	*cea*	Colicin E1	0	1 (4.2)	0	0
	*cia*	Colicin Ia	1 (4.2)	0	0	1 (4.2)
	*cib*	Colicin Ib	0	0	2 (8.3)	0
	*cma*	Colicin M	0	0	0	1 (4.2)
	*colE2*	Colicin E2	0	0	0	1 (4.2)
	*colE5*	Colicin E5 lysis protein Lys	0	2 (8.3)	0	0
	*cvaC*	Microcin C	1 (4.2)	0	0	0
	*eilA*	*Salmonella* HilA homolog	1 (4.2)	0	0	0
	*espA*	Type III secretions system	10 (41.7)	0	0	0
	*espB*	Secreted protein B	1 (4.2)	0	0	0
	*espC*	*Escherichia coli* enterotoxin EspC	1 (4.2)	0	0	0
	*espF*	EPEC secreted protein F; Type III secretion system	4 (16.7)	0	0	0
	*espI*	Serine protease autotransporters of *Enterobacteriaceae* (SPATE) EspI	0	1 (4.2)	0	1 (4.2)
	*espJ*	Prophage-encoded type III secretion system effector	5 (20.8)	0	0	0
	*espP*	Extracellular serine protease plasmid-encoded	0	0	0	1 (4.2)
	*espY2*	Non-LEE-encoded type III secreted effector	1 (4.2)	0	0	0
	*etpC*	EtpC Glycotransferase	2 (8.3)	0	0	0
	*etpD*	Type II secretion protein EtpD	1 (4.2)	0	1 (4.2)	0
	*etsC*	Putative type I secretion outer membrane protein	1 (4.2)	0	1 (4.2)	0
	*gad*	Glutamate decarboxylase	3 (12.5)	2 (8.3)	2 (8.3)	0
	*hha*	Hemolysin expression modulator Hha (previous *rmoA*)	5 (20.8)	1 (4.2)	0	0
	iss	Increased serum survival	4 (16.7)	2 (8.3)	1 (4.2)	2 (8.3)
	*kpsE*	Capsule polysaccharide export inner-membrane protein	0	0	0	1 (4.2)
	*mchB*	Precursor of microcin H47	0	1 (4.2)	0	1 (4.2)
	*mchC*	MchC protein	0	1 (4.2)	0	1 (4.2)
	*mchF*	ABC transporter protein MchF	1 (4.2)	1 (4.2)	0	1 (4.2)
	*mcmA*	Microcin M	0	1 (4.2)	0	1 (4.2)
	*neuC*	Polysialic acid capsule biosynthesis protein	0	2 (8.3)	0	0
	*nleB*	Non-LEE encoded effector B	6 (25)	0	0	0
	*ompT*	Outer membrane protease (protein protease 7)	3 (12.5)	0	1 (4.2)	2 (8.3)
	*ORF3*	Isoprenoid Biosynthesis	0	6 (25)	0	0
	*ORF4*	Putative isopentenyl-diphosphate delta-isomerase	0	6 (25)	0	0
	*shiA*	Homologue of the *Shigella flexneri* SHI-2 pathogenicity island gene *shiA*	0	2 (8.3)	1 (4.2)	0
	*shiB*	Homologue of the *Shigella flexneri* SHI-2 pathogenicity island gene *shiB*	1 (4.2)	3 (12.5)	0	1 (4.2)
	*tia*	Tia invasion determinant	0	0	0	1 (4.2)
	*traJ*	Protein TraJ (Positive regulator of conjugal transfer operon)	4 (16.7)	3 (12.5)	3 (12.5)	0
	*traT*	Outer membrane protein complement resistance	3 (12.5)	3 (12.5)	3 (12.5)	3 (12.5)
	*tsh*	Serine protease autotransporter of *Enterobacteriaceae* (SPATE)-Immunoglobulin A1 protease- Temperature-sensitive hemagglutinin	1 (4.2)	0	0	0
Iron uptake	*aalF*	CS23 Minor structural subunit	0	0	0	1 (4.2)
	*chuA*	Outer membrane hemin receptor	2 (8.3)	0	0	0
	*fyuA*	Yersiniabactin siderophore receptor	0	3 (12.5)	0	1 (4.2)
	*ireA*	Iron-regulated outer membrane protein IreA	0	2 (8.3)	0	0
	*iroN*	Salmochelin siderophore receptor protein	1 (4.2)	0	0	0
	*irp2*	Yersiniabactin non-ribosomal peptide synthetase	0	2 (8.3)	0	1 (4.2)
	*iucC*	Aerobactin synthetase	1 (4.2)	4 (16.7)	0	1 (4.2)
	*iutA*	Ferric aerobactin receptor	1 (4.2)	4 (16.7)	0	1 (4.2)
	*sitA*	Iron transport protein	1 (4.2)	2 (8.3)	1 (4.2)	0
	*terC*	Tellurium ion resistance protein	9 (37.5)	6 (25)	5 (20.8)	3 (12.5)
Toxins	*astA*	Heat-stable enterotoxin EAST-1	1 (4.2)	4 (16.7)	4 (16.7)	0
	*cdt-IIIB*	Cytolethal distending toxin III subunit B	0	0	0	1 (4.2)
	*cnf2*	Cytotoxic necrotizing factor 2	0	0	0	1 (4.2)
	*ehxA*	Enterohaemolysin	0	0	0	1 (4.2)
	*eltIAB-8*	Heat-labile enterotoxin LTIh-8	0	0	3 (12.5)	0
	*eltIAB-11*	Heat-labile enterotoxin LTIh-11				
	*estah-STa3*	Heat-stable enterotoxin STa3 human variant	0	0	2 (8.3)	0
	*hlyA*	Hemolysin A	1 (4.2)	0	0	0
	*hlyE*	Avian *E. coli* haemolysin	8 (33.3)	6 (25)	4 (16.7)	3 (12.5)
	*hlyF*	Hemolysin F	1 (4.2)	0	1 (4.2)	0
	*pet*	Autotransporter enterotoxin	1 (4.2)	0	0	0
	*pic*	Serine protease autotransporter of *Enterobacteriaceae* (SPATE) Pic	0	2 (8.3)	0	0
	*sat*	Serine protease autotransporter of *Enterobacteriaceae* (SPATE) Sat	2 (8.3)	2 (8.3)	0	0
	*senB*	Plasmid-encoded enterotoxin	2 (8.3)	0	0	0
	*sigA*	Serine protease autotransporter of *Enterobacteriaceae* (SPATE) *Shigella* IgA-like protease homologue	0	3 (12.5)	0	0
	*stx1*	Shiga toxin 1	0	0	0	2 (8.3)
	*stx2*	Shiga toxin 2	0	0	0	1 (4.2)
	*subA*	Subtilase toxin subunit	0	0	0	1 (4.2)
	*toxB*	Toxin B	1 (4.2)	0	0	0
	*vat*	Serine protease autotransporter of *Enterobacteriaceae* (SPATE) Vat	0	0	0	1 (4.2)

**Table 4:** Distribution of VAGs among different *E. coli* phylogroups as determined by WGS.

Virulence-Associated Genes and Their Encoded Proteins/Functions			Prevalence in Each Phylogenetic Group N (%), N = 24			
			A (*n* = 7)	B1 (*n* = 15)	B2 (*n* = 1)	E (*n* = 1)
Adhesins	*aap*	Dispersin, antiaggregation protein	2 (8.3)	4 (16.7)	0	0
	*afaA*	Transcriptional regulator	1 (4.2)	0	0	0
	*afaC*	Outer membrane usher protein	1 (4.2)	0	0	0
	*afaD*	Afimbrial adhesion	2 (8.3)	2 (8.3)	0	0
	*agg3A*	AAF/III major fimbrial subunit	1 (4.2)	2 (8.3)	0	0
	*agg3B*	AAF/III minor adhesin	1 (4.2)	2 (8.3)	0	0
	*agg3C*	Usher, AAF/III assembly unit	1 (4.2)	2 (8.3)	0	0
	*agg3D*	Chaperone, AAF/III assembly unit	1 (4.2)	2 (8.3)	0	0
	*agg4A*	AAF/IV major fimbrial subunit	0	2 (8.3)	0	0
	*agg4C*	Usher, AAF/IV assembly unit	0	2 (8.3)	0	0
	*aggA*	AAF/I major fimbrial subunit	1 (4.2)	0	0	0
	*aggC*	Usher, AAF/I assembly unit	1 (4.2)	0	0	0
	*aggD*	Chaperone, AAF/I assembly unit	1 (4.2)	0	0	0
	*aggR*	AraC transcriptional activator	2 (8.3)	4 (16.7)	0	0
	*cif*	Type III secreted effector	2 (8.3)	1 (4.2)	0	1 (4.2)
	*csgA*	Curli major subunit CsgA	7 (29.2)	15 (62.5)	1 (4.2)	1 (4.2)
	*cswR*	CS12 Transcriptional activator	0	2 (8.3)	0	0
	*eaeA*	Intimin	3 (12.5)	4 (16.7)	1 (4.2)	1 (4.2)
	*efa1*	EHEC factor for adherence; lymphostatin Efa1-LifA	0	1 (4.2)	0	0
	*etpB*	EtpB nonfimbrial adhesin/TPS transporter	1 (4.2)	0	0	0
	*faeC*	F4 (K88) Minor fimbrial subunit	0	1 (4.2)	0	0
	*fdeC*	Intimin-like adhesin FdeC	0	12 (50)	0	1 (4.2)
	*fimH*	Type 1 fimbriae	6 (25)	13 (54.2)	0	1 (4.2)
	*hra*	Heat-resistant agglutinin	1 (4.2)	2 (8.3)	0	0
	*iha*	Adherence protein	0	5 (20.8)	0	0
	*lpfA*	Long polar fimbriae	0	14 (58.3)	0	0
	*nlpI*	Lipoprotein NlpI precursor	6 (25)	12 (50)	1 (4.2)	1 (4.2)
	*perA*	EPEC adherence factor	0	0	1 (4.2)	0
	*tir*	Translocated intimin receptor protein	3 (12.5)	5 (20.8)	0	1 (4.2)
	*yehA*	Outer membrane lipoprotein, YHD fimbrial cluster	7 (29.2)	7 (29.2)	1 (4.2)	0
	*yehB*	Usher, YHD fimbrial cluster	5 (20.8)	15 (62.5)	1 (4.2)	1 (4.2)
	*yehC*	Chaperone, YHD fimbrial cluster	5 (20.8)	15 (62.5)	1 (4.2)	1 (4.2)
	*yehD*	Major pilin subunit, YHD fimbrial cluster	5 (20.8)	14 (58.3)	1 (4.2)	1 (4.2)
	*yfcV*	Fimbrial protein	0	0	1 (4.2)	1 (4.2)
Protectins	*aaiC*	Type VI secretion protein	1 (4.2)	1 (4.2)	0	0
	*aar*	AggR-activated regulator	1 (4.2)	1 (4.2)	0	0
	*aatA*	Dispersin transporter protein	1 (4.2)	2 (8.3)	0	0
	*aamR*:FN554766	Not defined	0	1 (4.2)	0	0
	*anr*	AraC negative regulator	4 (16.7)	2 (8.3)	0	0
	*aslA*	Arylsulfatase-like protein	1 (4.2)	0	1 (4.2)	0
	*capU*	Hexosyltransferase homolog	1 (4.2)	3 (12.5)	0	0
	*cba*	Colicin B	0	1 (4.2)	0	0
	*cea*	Colicin E1	1 (4.2)	0	0	0
	*cia*	Colicin Ia	1 (4.2)	1 (4.2)	0	0
	*cib*	Colicin Ib	2 (8.3)	0	0	0
	*cma*	Colicin M	0	1 (4.2)	0	0
	*colE2*	Colicin E2	0	1 (4.2)	0	0
	*colE5*	Colicin E5 lysis protein Lys	0	2 (8.3)	0	0
	*cvaC*	Microcin C	1 (4.2)	0	0	0
	*eilA*	*Salmonella* HilA homolog	0	0	0	1 (4.2)
	*espA*	Type III secretions system	3 (12.5)	5 (20.8)	1 (4.2)	1 (4.2)
	*espB*	Secreted protein B	0	1 (4.2)	0	0
	*espC*	*Escherichia coli* enterotoxin EspC	0	0	1 (4.2)	0
	*espF*	EPEC secreted protein F; Type III secretion system	1 (4.2)	3 (12.5)	0	0
	*espI*	Serine protease autotransporter of *Enterobacteriaceae* (SPATE) EspI	1 (4.2)	1 (4.2)	0	0
	*espJ*	Prophage-encoded type III secretion system effector	0	3 (12.5)	1 (4.2)	1 (4.2)
	*espP*	Extracellular serine protease plasmid-encoded	0	1 (4.2)	0	0
	*espY2*	Non-LEE-encoded type III secreted effector	0	0	0	1 (4.2)
	*etpC*	EtpC Glycotransferase	1 (4.2)	0	0	0
	*etpD*	Type II secretion protein EtpD	1 (4.2)	1 (4.2)	0	0
	*etsC*	Putative type I secretion outer membrane protein	1 (4.2)	0	0	0
	*gad*	Glutamate decarboxylase	2 (8.3)	4 (16.7)	0	0
	*hha*	Hemolysin expression modulator Hha (previous *rmoA*)	1 (4.2)	3 (12.5)	0	1 (4.2)
	iss	Increased serum survival	3 (12.5)	5 (20.8)	0	0
	*kpsE*	Capsule polysaccharide export inner-membrane protein	0	1 (4.2)	0	0
	*mchB*	Precursor of microcin H47	0	2 (8.3)	0	0
	*mchC*	MchC protein	0	2 (8.3)	0	0
	*mchF*	ABC transporter protein MchF	1 (4.2)	2 (8.3)	0	0
	*mcmA*	Microcin M	0	2 (8.3)	0	0
	*neuC*	Polysialic acid capsule biosynthesis protein	0	1 (4.2)	0	0
	*nleB*	Non-LEE encoded effector B	3 (12.5)	2 (8.3)	0	1 (4.2)
	*ompT*	Outer membrane protease (protein protease 7)	1 (4.2)	3 (12.5)	1 (4.2)	0
	*ORF3*	Isoprenoid Biosynthesis	2 (8.3)	4 (16.7)	0	0
	*ORF4*	Putative isopentenyl-diphosphate delta-isomerase	2 (8.3)	4 (16.7)		0
	*shiA*	Homologue of the *Shigella flexneri* SHI-2 pathogenicity island gene *shiA*	2 (8.3)	1 (4.2)	0	0
	*shiB*	Homologue of the *Shigella flexneri* SHI-2 pathogenicity island gene *shiB*	1 (4.2)	4 (16.7)	0	0
	*tia*	Tia invasion determinant	0	1 (4.2)	0	0
	*traJ*	Protein TraJ (Positive regulator of conjugal transfer operon)	4 (16.7)	3 (12.5)	1 (4.2)	0
	*traT*	Outer membrane protein complement resistance	3 (12.5)	6 (25)	0	0
	*tsh*	Serine protease autotransporter of *Enterobacteriaceae* (SPATE)-Immunoglobulin A1 protease- Temperature-sensitive hemagglutinin	1 (4.2)	0	0	0
Iron uptake	*aalF*	CS23 Minor structural subunit	0	1 (4.2)	0	0
	*chuA*	Outer membrane hemin receptor	0	0	1 (4.2)	1 (4.2)
	*fyuA*	Yersiniabactin siderophore receptor	2 (8.3)	2 (8.3)	0	0
	*ireA*	Iron-regulated outer membrane protein IreA	0	1 (4.2)	0	0
	*iroN*	Salmochelin siderophore receptor protein	1 (4.2)	0	0	0
	*irp2*	Yersiniabactin non-ribosomal peptide synthetase	1 (4.2)	2 (8.3)	0	0
	*iucC*	Aerobactin synthetase	1 (4.2)	4 (16.7)	0	0
	*iutA*	Ferric aerobactin receptor	1 (4.2)	4 (16.7)	0	0
	*sitA*	Iron transport protein	1 (4.2)	1 (4.2)	0	0
	*terC*	Tellurium ion resistance protein	7 (29.2)	15 (62.5)	1 (4.2)	1 (4.2)
Toxins	*astA*	Heat-stable enterotoxin EAST-1	3 (12.5)	4 (16.7)	0	0
	*cdt-IIIB*	Cytolethal distending toxin III subunit B	0	1 (4.2)	0	0
	*cnf2*	Cytotoxic necrotizing factor 2	0	1 (4.2)	0	0
	*ehxA*	Enterohaemolysin	0	1 (4.2)	0	0
	*eltIAB-8*	Heat-labile enterotoxin LTIh-8	0	1 (4.2)	0	0
	*eltIAB-11*	Heat-labile enterotoxin LTIh-11	0	2 (8.3)	0	0
	*estah-STa3*	Heat-stable enterotoxin STa3 human variant	2 (8.3)	0	0	0
	*hlyA*	Hemolysin A	0	1 (4.2)	0	0
	*hlyE*	Avian *E. coli* haemolysin	6 (25)	15 (62.5)	0	1 (4.2)
	*hlyF*	Hemolysin F	1 (4.2)	0	0	0
	*pet*	Autotransporter enterotoxin	0	1 (4.2)	0	0
	*pic*	Serine protease autotransporter of *Enterobacteriaceae* (SPATE) Pic	1 (4.2)	1 (4.2)	0	0
	*sat*	Serine protease autotransporter of *Enterobacteriaceae* (SPATE) Sat	1 (4.2)	3 (12.5)	0	0
	*senB*	Plasmid-encoded enterotoxin	0	2 (8.3)	0	0
	*sigA*	Serine protease autotransporter of *Enterobacteriaceae* (SPATE), *Shigella* IgA-like protease homologue	0	2 (8.3)	0	0
	*stx1*	Shiga toxin 1	0	2 (8.3)	0	0
	*stx2*	Shiga toxin 2	0	1 (4.2)	0	0
	*subA*	Subtilase toxin subunit	0	1 (4.2)	0	0
	*toxB*	Toxin B	0	0	1 (4.2)	0
	*vat*	Serine protease autotransporter of *Enterobacteriaceae* (SPATE) Vat	0	1 (4.2)	0	0

**Table 5:** Antimicrobial resistance genes among DEC isolates.

Antimicrobial Class	Antimicrobial Resistance Gene (ARG)	Prevalence N (%)	Conferring Resistance to	Found in DEC Pathotype
β-lactams	*bla*_TEM-1B_	12 (50)	amoxicillin, ampicillin, piperacillin, ticarcillin, cephalothin	EPEC, EAEC, ETEC
	*bla*_TEM-122_	1 (4.2)	amoxicillin, amoxicillin + clavulanic acid, ampicillin, ampicillin + clavulanic acid, piperacillin, piperacillin + tazobactam, ticarcillin, ticarcillin + clavulanic acid	ETEC
	*bla*_TEM-163_	1 (4.2)	amoxicillin, amoxicillin + clavulanic acid, ampicillin, ampicillin + clavulanic acid, piperacillin, piperacillin + tazobactam, ticarcillin, ticarcillin + clavulanic acid	EPEC
	*bla*_CTX-M-3_	1 (4.2)	amoxicillin, ampicillin, cefepime, cefotaxime, ceftazidime, piperacillin, aztreonam, ticarcillin, ceftriaxone	EPEC
	*bla*_CTX-M-15_	2 (8.3)	amoxicillin, ampicillin, cefepime, cefotaxime, ceftazidime, piperacillin, aztreonam, ticarcillin, ceftriaxone	ETEC
Sulfonamides	*sul1*	2 (8.3)	sulfamethoxazole	EPEC
	*sul2*	8 (33.3)	sulfamethoxazole	EPEC, EAEC, ETEC
Aminoglycosides	*aph(6)-Id*	6 (25)	streptomycin	EPEC, ETEC
	*aph(3″)-Ib*	6 (25)	streptomycin	EPEC, ETEC
	*aac(3)-IIa*	1 (4.2)	gentamicin, tobramycin	EPEC
	*aadA1*	2 (8.3)	streptomycin, spectinomycin	EAEC, ETEC
	*aadA5*	1 (4.2)	streptomycin, spectinomycin	EPEC
	*aadA24*	1 (4.2)	streptomycin, spectinomycin	EPEC
Tetracyclines	*tet*(A)	4 (16.7)	tetracycline, doxycycline	EPEC
	*tet*(B)	3 (12.5)	tetracycline, doxycycline, minocycline	EPEC, ETEC
Quinolones	*qnrS1*	2 (8.3)	ciprofloxacin	ETEC
Trimethoprim	*dfrA1*	2 (8.3)	trimethoprim	EAEC, ETEC
	*dfrA7*	1 (4.2)	trimethoprim	EPEC
	*dfrA8*	3 (12.5)	trimethoprim	EPEC, EAEC, ETEC
	*dfrA14*	1 (4.2)	trimethoprim	EPEC
	*dfrA15*	1 (4.2)	trimethoprim	ETEC
	*dfrA17*	1 (4.2)	trimethoprim	EPEC
Macrolide/lincosamide/streptogramin (MLS) group	*mph*(A)	6 (25)	erythromycin, azithromycin, spiramycin, telithromycin	EPEC, EAEC, ETEC
	*erm*(B)	1 (4.2)	lincomycin, clindamycin, erythromycin, quinupristin, pristinamycin IA, virginiamycin S	EAEC

## Data Availability

The generated sequencing raw data and assembled genomes were submitted to SRA—Sequence Read Archive (accession number: PRJNA1105046, URL https://www.ncbi.nlm.nih.gov/bioproject/PRJNA1105046, accessed on 10 May 2024).

## Supplementary Materials

The following supporting information can be downloaded at: https://www.mdpi.com/article/10.3390/ijms251910251/s1.
